# Non-pharmacological interventions a feasible option for addressing dementia-related sleep problems in the context of family care

**DOI:** 10.1186/s40814-021-00851-x

**Published:** 2021-05-26

**Authors:** Rosemary Gibson, Anthony Dowell, Linda Jones, Philippa Gander

**Affiliations:** 1 Sleep/Wake Research Centre, Massey University, Private Bag 756, Wellington, New Zealand; 2 Primary Health Care and General Practice University of Otago, Wellington, New Zealand; 3 School of Psychology, Massey University, Wellington, New Zealand

**Keywords:** Actigraphy, Ageing, Caregiving, Dementia, Sleep problems, Non-pharmacological interventions

## Abstract

**Background:**

Sleep disturbances are challenging symptoms associated with mild cognitive impairment or dementia (MCIoD). This study assessed the feasibility of sleep monitoring and non-pharmacological interventions to improve the sleep of New Zealanders with MCIoD and their family carers.

**Methods:**

A 5-week multi-modal intervention consisting of timed bright light therapy, physical activity, and sleep education was piloted. Sleep was monitored for a week at baseline and conclusion of the trial using actigraphy, diaries, and questionnaires alongside additional health and wellbeing information concerning both care recipients and carers.

**Results:**

Fifteen pairs participated, 9 completed the trial. Patterns of attrition and participant feedback are discussed. Case studies showed that six of the care recipients had minor improvements to sleep efficiency. Some also had improved subjective sleep ratings and quality of life. Changes did not clearly translate to family carers. However, five of them also showed some improvements in sleep status and mental health. Health deterioration of care recipients may mask the effects of the intervention.

**Conclusions:**

It is feasible to use non-pharmacological sleep interventions for people with MCIoD and their family carers. Given the limited treatment options, further consideration of such interventions in future research and clinical practice is warranted.

**Trial registration:**

As this study was to assess the feasibility of proposed methods, it was an observational study without case-control groups nor a medical-based intervention, clinical registration was not required. A future full version of the trial would be registered with the Australian New Zealand Clinical Trails Registry.

## Key messages regarding feasibility

- It was uncertain whether participants could be recruited and retained over the trial period in the community as opposed to an institutionalised setting. It was also uncertain whether the intervention and methods of assessment would be acceptable to people with dementia and their carers living in the community.
- Participants rated the study methods and interventions as feasible and easy to use accept the sleep diary which, for some, was reported as onerous. Compliance was greater for the timed light aspect on the intervention rather than the physical activity. Participant retention was impacted by degrees of sleep problems and cognitive impairment as well as admittance into hospital or care facilities.
- Recommendations are made around tailoring a trial towards those most in need (i.e. poorer sleep and limited access to bright light and physical activity) and using a person-centred approach to engage and deliver such intervention. Also, that actigraphy monitoring may be more acceptable and reliable without concurrent sleep diaries.

## Introduction

Disordered, irregular sleep, and sleep deprivation have been associated with poorer cognition, physical and mental health, and mortality [1, 2]. Sleep deterioration, with ageing, has been related to multiple physiological, psychological, and health factors [3, 4]. As life expectancy rises, cases of mild cognitive impairment and dementia are increasing [5]. Sleep disturbances often occur in the early stages of cognitive decline and have been identified as among the most challenging symptoms associated with progressive dementias (such as Alzheimer’s disease) [6, 7]. These disturbances have been related to reduced activity and function of the circadian master clock in the hypothalamus, as well as psycho-social and behavioural factors [6]. Not surprisingly, the sleep of family carers is also often affected [8]. Situations where a person with mild cognitive impairment or dementia (MCIoD) and their family carer are both sleeping poorly tend to be those which are more difficult to manage [8, 9].

Pharmacological options for the symptoms of MCIoD (including sleep disturbances) often have side effects and limited effectiveness [10]. There is great emphasis on exploring non-pharmacological strategies to modify and manage the symptoms. Better management of sleep health could lead to improvements in waking symptoms and wellbeing for people with MCIoD as well as family carers. Interventions using timed bright light and/or physical activity can help stimulate the circadian system whilst promoting overall sleep health [11, 12]. Previous studies have found that interventions combining bright light therapy with physical or social activities as well as sleep hygiene education can promote more robust sleep timing and less disturbed sleep among people with dementia [11–14]. However, community-based trials are lacking and the feasibility of conducting such research in New Zealand (NZ) has yet to be explored. The NZ context differs from previous studies with regards to population size, cultural diversity, as well as sleep and age-related healthcare services. Action plans focus on supporting families affected by dementia to live their best possible lives [15]. Often, there is desire to remain at home under the support of family; this is particularly the case among older Māori (the indigenous people of NZ) [16]. Research to understand and meet such objectives are growing but, to date, sleep health is yet to be addressed. Pilot data are required before designing and implementing further trials among NZ’s diverse populations.

## Aims and objectives

1. To pilot a community-based approach for gathering objective and subjective data on the sleep of people with MCIoD and their family carer in NZ.
2. To design and examine the feasibility of a trial for testing non-pharmacological interventions to improve sleep and waking function.

Feasibility outcomes included the acceptability of the protocol and usability of measures as well as positive impacts of the intervention. These were measured by assessing the quality of data, changes across the intervention, as well as gathering feedback both during weekly phone conversations and using formal feedback questionnaires post trial. Success of feasibility was gauged on a case-by-case basis by subjective responses as well as the objective sleep and wellbeing measures. Feedback was used to identifying the most useful measures, appropriate outcome variables, and factors affecting participant compliance and withdrawal (see “Data analysis” section for more details).

## Methods

### Participants

Fifteen pairs including a person with MCIoD (“care recipients”) and their family carers (“carers”) were recruited via Alzheimers Wellington (a local organisation offering support and advocacy services for families affected by dementia) as well as via community advertisements, presentations, and referrals from healthcare professionals. Due to the pilot nature of the study, convenience sampling was used. Inclusion criteria were living together in the Wellington community, the care recipient being aged ≥ 65 years, and that they and/or their carer had an interest in improving their sleep. Due to the underdiagnoses of dementia [5], status of MCIoD was based on participants’ report and involvement with dementia-related services. Exclusion criteria were having eye conditions or taking medications which could cause photosensitivity to the light therapy element of the protocol [17].

Written consent was gathered from each pair. Verbal assent was also recorded during the study briefing, to offer protection and/or an alternative for those with reduced cognitive capacity. Participant’s doctors were informed and given the opportunity to notify the researcher of any further contraindications for bright light exposure or contact the research team with any concerns.

### Materials

Sleep/wake activity of the pairs was measured for 7 days at baseline (Time 1) and during the fifth week of the intervention (Time 2) using the Actiwatch-2 (Mini Mitter, Philips, Respironics) worn on the non-dominant wrist. The Actiwatch-2 has a button serving as an event marker for participants to press at relevant times (e.g. at bedtime), and a sensor for measuring the amount and duration of ambient white light exposure in units of lux (from the wrist). Activity counts were recorded in 1-min intervals and the records were analysed using custom software provided by the manufacturer, using the medium sensitivity threshold. Time in bed was defined via the event marker and light data from the Actiwatch-2 and a sleep diary in the form of 24-h timelines. Participants recorded the times of sleep start and end for any sleep that was 10 min or longer. Diaries for the care recipients were maintained throughout the intervention to collect information on compliance.

Questionnaires were completed by the pairs at Times 1 and 2. These included demographic information as well as validated questionnaires concerning sleep [18, 19], health [20, 21], symptoms, and quality of life of those with CIoD [22–24] and the impact of care [25] (see Table 1). Open-ended questions also prompted feedback on the protocol and ratings of each aspect of the methods and intervention. This information was evaluated regardless of whether a pair completed the entire protocol.

**Table 1:** Content of questionnaires completed by care recipients and carers

		Completed by:	
Measure	Reference	Care recipient	Carer
Demographics	Age, gender, type of diagnosis, caregiving timing, and relief	✓	✓
Sleep timing and quality	Pittsburgh Sleep Quality Index (PSQI) range = 0–21, > 5 = “disturbed sleep”] [18]	✓	✓
Dementia-related sleep disturbance	Sleep Disorders Inventory (SDI) range = 0–12/”severely disturbed sleep” [19]		✓
Health	Self-Administered Comorbidity Questionnaire (SCQ [20]^)^ and medication list	✓	✓
Cognitive impairment	Mini-Mental State Examination (MMSE) range = 30–0, > 23 = “no impairment,” 19–23 = “mild cognitive impairment,” 10–18 = “moderate cognitive impairment,” and ≤ 9 = “severe cognitive impairment” [22]	✓	
Dementia-related symptoms	Revised Memory and Behaviour Problems Checklist (RMBPC) global score (0–96) plus sub-scores for memory (range = 0–28), depression (range = 0–36), and disruptive behaviours (range 0–32) with higher scores indicating increased frequency of behaviours and negative carer affect [23]		✓
Quality of life of recipient	Quality of Life in Alzheimer’s Disease: Patient and Caregiver Report (QOL-AD) range = 52 (excellent)—13 (poor) [24]	✓	✓
Mental health	Hospital Anxiety and Depression Scale (HAD), range 0–21, 0–7 = “normal,” 8–10 = “borderline,” 11–21 = heightened risk [21]		✓
Impact of caregiving	Carers of Older People in Europe index (COPE) range for positive impact of caring items = 15–0(< 12 = reduced coping). Range for negative impact of caring items = 0–18 (> 12 = reduced coping) [25]		✓

### Procedure

**Fig. 1:**
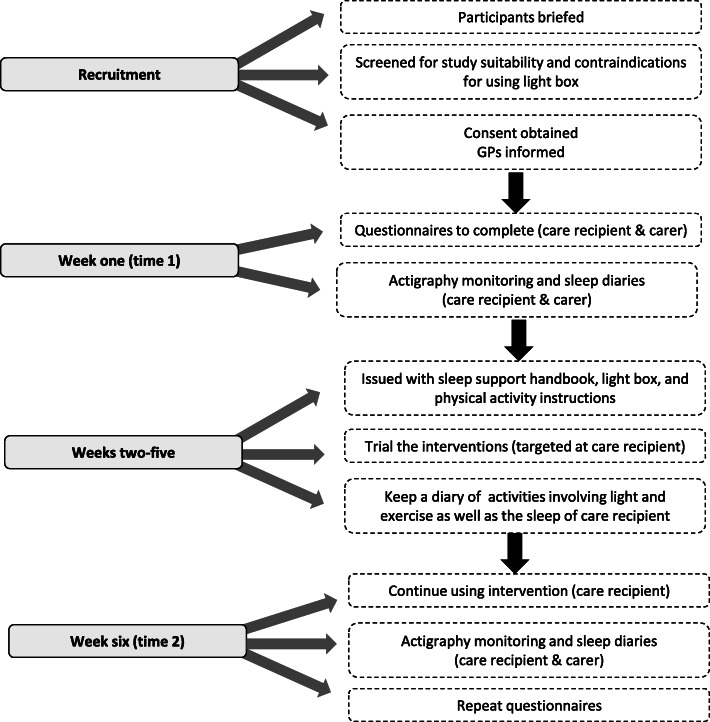
Overview of the study protocol

Figure 1 provides an overview of study procedures. Questionnaires were completed by the care recipient in the presence of the researcher and carer, to support completion. In cases when they were not cognitively able, the carer acted as a proxy. The interventions included timed bright light therapy, exercise, and sleep hygiene education. A day-light light box was provided (Uplift Technologies Inc., 2009) as an alternative to natural light. This provides up to 10,000 lux of illumination at a comfortable sitting distance (30–33 cm) [17]. The proposed exercise regime was 30–40 min of walking or yoga style stretches and movement (participants were loaned a senior’s exercise DVD and were given information about safe home exercise). A ‘Sleep Support Handbook’ was developed for the study that contained information on sleep with ageing and dementia, and sleep hygiene guidelines.

During the 5-week trial, care recipients were encouraged to get 30 min of bright light exposure every morning (or on as many mornings as possible) between 9 and 11 am, using either the light box or natural light. They were also asked to undertake the low-intensity exercises on as many days as possible during the middle of the day (11am–2pm). Carers were encouraged to join in with physical activities or supervise as they saw necessary. Finally, participants were encouraged to try some of the ideas/solutions offered in the handbook, and to note in the sleep diaries instances when they had tried new things. Participants were telephoned by the researcher on a weekly basis to remind them of the study protocol, gauge feasibility outcomes, and answer any questions regarding the study. They also had access to a free phone number to contact the researcher throughout for advice or assistance.

### Data analysis

Actigraphy records were analysed with the diary data to identify rest periods. Fifty percent of records were double-scored and anomalous responses and outliers were checked (average agreement rate = 84.8% for the care recipient records, 92.1% for carers). Sections of complete inactivity (due to the actiwatch not being worn or for unknown reasons) were excluded. Rest periods were categorised into night and day. Night was defined as the time the participant initially went to bed intending to sleep (bedtime) until the final morning rising (rise time). Standard actigraphy variables were derived from the software (e.g. total sleep time, sleep efficiency, number of awakenings) and compared for each participant between Times 1 and 2. Global scores were calculated for each validated questionnaire and its subscales. For scales with established threshold values, participants were categorised according to whether they scored above or below the thresholds. The Pittsburgh Sleep Quality Index (PSQI) was key, with scores above 5 indicating problematic sleep [18].

Compliance with the intervention was estimated from the diaries (calculated from the number of days with light or activity logged within and outside of the requested time frames) as well as by comparing Time 1 light (9am–11am) and activity data (11am–2pm) from the Actiwatch 2’s to Time 2 data. Descriptive statistics from the nominal questions in the feedback form were calculated and field notes from home visits and telephone conversations collated. Due to the small and heterogeneous sample, the effect and feasibility of the protocol and intervention was based on case studies [26]. Individual scores were plotted for within-subject comparisons between Times 1 and 2, and to visually compare the distribution of results between participants who completed the trial versus those who did not.

## Results

Fifteen pairs took part. The demographic details of the care recipients and their study outcome are in Table 2. All but two pairs were spouses (opposite-gender couples). Pairs 6 and 14 were parent and child. Due to the small convenience sample, ethnicity was not recorded. The majority (*n* = 11) of the carers were not in paid employment. However, four were also working 20–40 h per week (ID’s 5, 6, 8, and 14). Summary data for key questionnaire and actigraphic data are provided in Table 3. This shows that care recipients had poorer and more variable objective sleep duration and efficiency compared to carers. In contrast, care recipients’ PSQI sores generally indicated less subjective sleep disturbance than carers.

**Table 2:** Brief demographic details with outcome summaries

ID	Age	Gender	Diagnosis	MMSE	Carer age	Outcome summary
Those who completed:						
1	73	Male	VD	15	73	Dementia-related deterioration, minor improvements to care recipient’s PSQI and actigraphic sleep
2	82	Male	VD	13	74	Dementia-related deterioration, PSQI improved for both
3	72	Male	AD	14	71	Good quality sleep at onset, minor improvement to care recipient’s PSQI
4	76	Male	Undefined	23	74	Good quality sleep at onset, no improvements observed, care recipient had marked daytime sleepiness
5	82	Male	LBD	5	68	Marked improvements to both subjective and actigraphic sleep variables as well as waking function of care recipient
6	83	Female	Undefined	27	46	Minor subjective and actigraphic improvements to sleep, however carer reported deterioration to care recipient’s sleep
7	73	Female	AD	16	78	Good quality sleep at onset, no improvements observed
8	66	Male	AD	18	67	Good sleep quality at onset, no improvements observed. Struggled with protocol
9	80	Male	VD	7	54	Minor improvements to actigraphic sleep of both. Carer reported stress and sleep disturbed by care recipient
Those who withdrew:						
10	80	Female	AD	21	81	Withdrew due to care recipient moving into care home at end of week 1
11	72	Male	AD	13	68	Withdrew as care recipient admitted to hospital
12	88	Male	MCI	29	75	Withdrew after losing motivation after period of respite care
13	83	Female	VD	20	82	Withdrew at the end of week 1, sleep not considered problematic enough
14	89	Female	Undefined	23	55	Withdrew as intervention protocol considered too stressful
15	73	Male	VD	28	55	Withdrew during week 1 due to care recipient moving into care facility after a fall

*Abbreviations*: *VD* vascular dementia, *AD* Alzheimer’s disease, *LBD* Lewy body dementia, *MCI* mild cognitive impairment, *undefined* type of dementia unknown/not specified in diagnosis, *MMSE* Mini-Mental Status Exam, *PSQI* Pittsburgh Sleep Quality Index

**Table 3:** Descriptive statistics and paired summaries of key variables for participants who started and completed the protocol (first and second line for each variable respectively)

	Time 1			Time 2		
Variable (range of scale)	N	Median	(range)	N	Median	(range)
**Care recipient**						
Sleep duration night, hours	15	8.0	(5.5–10.6)			
	9	8.1	(6.7–9.8)	9	8.2	(5.7–12.3)
Sleep efficiency night (%)	15	85.0	(61.3–98.4)			
	9	85.0	(62.0–95.9)	9	85.4	(61.4–96.5)
Sleep duration day, minutes	15	47.0	(0.0–78.0)			
	9	51.5	(0.0–78.0)	9	29.5	(0.0–192.0)
PSQI (0–21)	15	4.0	(1.0–13.0)			
	9	4.0	(1.0–8.0)	9	3.0	(2.0–7.0)
SDI-AD (0–12)	14	0.2	(0.0–4.2)			
	9	0.9	(0.0–4.0)	9	1.1	(0.0–3.0)
MMSE (30–0)	14	18.0	(7.0–29.0)			
	9	15.0	(5.0–27.0)	9	15.0	(6.0–26.0)
RMBPC Behaviour frequency (0–32)	15	3.5	(0.0–11.0)			
	9	3.0	(0.0–11.0)		5.0	(2.0–10.0)
RMBPC Behaviour reaction (0–32)	15	3.5	(0.0–19.8)			
	9	3.3	(0.0–12.0)	9	3.5	(0.0–24.0)
QOL-AD (CR rated), (52–13)	14	39.0	(24.0–48.0)			
	8	39.5	(24.0–45.0)	9	36.0	(18.0–49.0)
QOL-AD (C rated), (52–13)	15	30.0	(23.0–40.0)			
	9	30.0	(23.0–40.0)	9	34.0	(22.0–40.0)
**Carer**						
Sleep duration night, hours	14	7.5	(5.9–8.9)			
	8	7.9	(6.1–8.9)	7	7.6	6.7–9.1
Sleep efficiency (% sleep night)	14	89.6	(72.5–93.7)			
	8	90.0	(85.1–93.7)	7	87.1	(83.3–92.4)
Sleep duration day, minutes	14	7.0	(0.0–80.0)			
	8	0.0	(0.0–80.0)	7	0.0	(0.0–47.5)
PSQI (0–21)	14	8.0	(1.0–10.0)			
	8	8.0	(1.0–9.0)	9	5.0	(2.0–9.0)
COPE positive (15–0)	15	11.0	(4.0–14.0)	9	10.0	(4.0–15.0)
	9	10.0	(4.0–14.0)			
COPE negative (0–18)	15	5.0	(1.0–12.0)	9	10.0	(0.0–12.0)
	9	6.0	(1.0–12.0)			
HADS-Anxiety (0–21)	15	4.0	(0.0–12.0)	9	6.0	(1.0–12.0)
	9	4.0	(0.0–12.0)			
HADS-Depression (0–21)	15	4.0	(0.0–9.0)	9	5.0	(0.0–11.0)

*PSQI* Pittsburgh Sleep Quality Index, *SDI-AD* Sleep Disorders Inventory Alzheimer’s Disease, *MMSE* Mini Mental Status Exam, *RMBPC* Revised Memory Behaviour Problem Checklist (global scores unavailable due to missing data), *QOL-AD* Quality of Life in Alzheimer’s disease” Patient and Caregiver report, *COPE* Carers of Older People in Europe index, *HADS* Hospital Anxiety and Depression Scale

Six of the pairs withdrew during the trial. Care recipients in these pairs tended to be less cognitively impaired (median MMSE = 20.5, range = 13.0–29.0) compared to those who completed the trial (median MMSE = 15.5, range = 7.0–27.0). However, the RMBPC scores indicated that those who withdrew tended to have more frequent and disruptive behaviours (median = 6.3, range = 0.0–10.5 vs. median = 3.0, range = 0–11). The carers reaction rating to these behaviours was also higher among those who withdrew (median = 5.8, range = 0–19.8) compared to those who completed the trial (median = 3.0, range = 0–12). Two withdrawal “types” were identified among the care recipients. Firstly, those who had less severe symptoms of dementia with minor sleep problems and lost interest or were confused by the study protocol (pairs 12, 13, and 14); and secondly, those who had more advanced symptoms of dementia with substantial sleep problems but were admitted to hospital or a care facility during the trial period and so could not complete the trial (pairs 10, 11, and 15). All pairs rated the actigraphy and questionnaires as ok-easy to use. Only one rated the diary as difficult, but some carers noted the challenge of being able to reliably document sleep and wake bouts across the 24-h day for care recipients who had very fragmented sleep. For those who completed the trial (*n* = 9 pairs), diary-reported compliance with the bright light therapy ranged from 37 to 97% of the days during the 5-week intervention (median 72%). Compliance with the exercises was lower (3–86% days, median 55%). The interventions were not always used in the specified timeframes (56% of the time and 38% of the time respectively). The Actiwatch 2 data showed an 85% increase in mean light exposure for care recipients within the 9am–11am timeframe between baseline and trial completion (mean T1 = 4905 lux, SD = 5377 lux vs. mean T2 = 9077, SD = 14,709) compared to just a 2% increase in physical activity counts within the 11am–2pm timeframe (mean activity counts T1 = 27,635, SD = 15,096 vs. mean activity counts T2 = 28,181, SD = 13,338). There was no clear pattern of baseline measures being reliable predictors for trial outcomes. Feedback data revealed that none of the pairs found the light box difficult to use. However, two found the exercise DVD difficult and one reported difficulty with the education booklet. Seven of the nine said that they would recommend the interventions to others (the others said they maybe would).

**Fig. 2:**
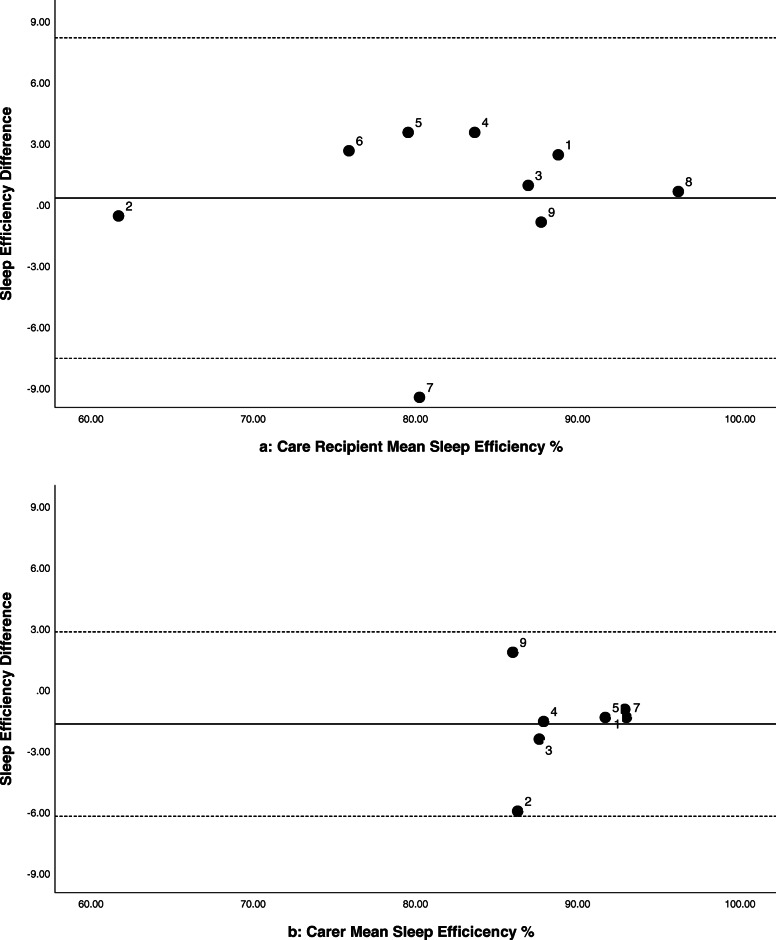
Bland-Altman plots comparing sleep efficiency at night between Times 1 and 2 for care recipients 2(**a**) and family carers 2(**b**)

**Fig. 3:**
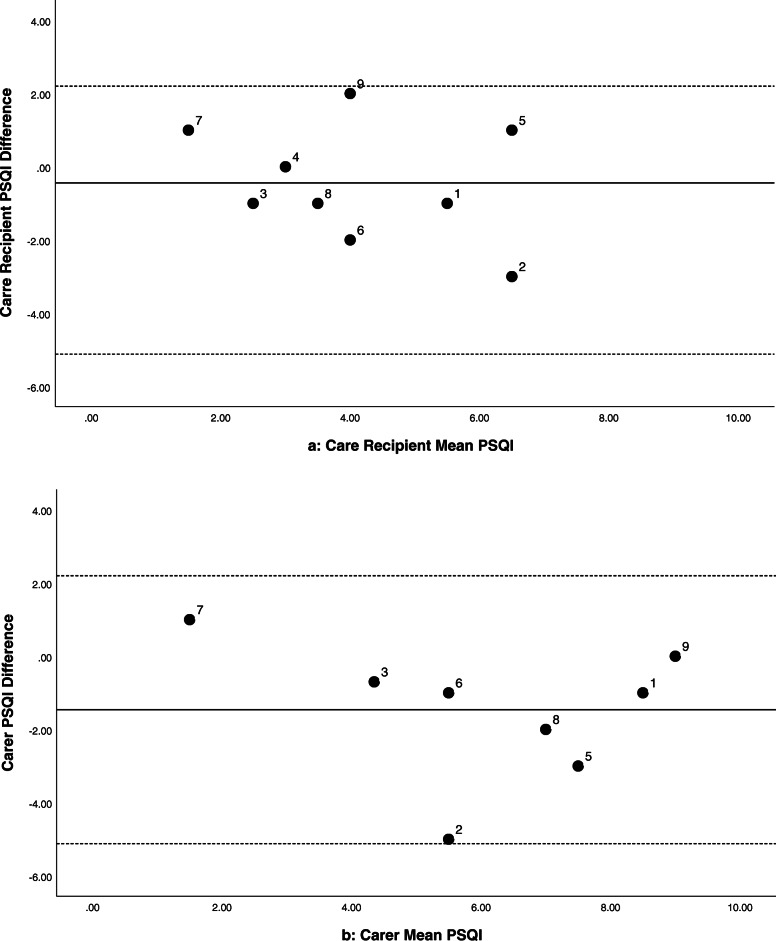
Bland-Altman plots comparing PSQI scores between Times 1 and 2 for care recipients (3**a**) and family carers (3**b**)

Six of the care recipients showed minor improvements in their sleep efficiency at night (0.6–3.6%, see Fig. 2a). Their median time in bed was 10.2 h (range = 8.7–11.9 h), indicating an increase of 4–22 min more sleep at Time 2. While the grouped data are variable, there were cases with more marked improvements in sleep and wellbeing (e.g. Participant 5, case study presented elsewhere) [26]. The majority (six) of the care recipients scored normal/good on the PSQI at Time 1 (< 5). Nevertheless, five showed 1–3 points of improvement on their PSQI scores at Time 2 (see Fig. 3a). Actigraphic and subjective improvements in sleep (PSQI or SDI scores) were not clearly related. Of the five care recipients who had improved PSQI ratings at Time 2, two also had improved scores on the MMSE (3–4 points) and three had improvements to their quality of life as rated by their carer QOL-AD (2–7 points). However, between Times 1 and 2, six had reductions of 1–7 points in their MMSE score. Furthermore, many of the behavioural symptoms associated with dementia (as indicated by participant feedback and scores on the RMBPC or QOL-AD) deteriorated across the trial period. Six out of the nine carers had PSQI scores that indicated disturbed sleep (> 5) at Time 1. Five had 1–5 points of improvement in their PSQI scores between Times 1 and 2 (see Fig. 3b). Of these five, three also had decreased depression scores at Time 2 (as indicated by the HADS). However, the majority (seven) scored within the normal range for symptoms of anxiety and depression at Time 1. Improvements to the care recipient’s sleep did not always translate into improved sleep, mood, or coping of their family carer (Figs. 2b and 3b).

## Discussion

This study indicates that a combined intervention using bright light therapy and/or therapeutic exercise with sleep hygiene education is feasible for people with MCIoD and their family carers at home. Given the paucity of options for treating dementia, the intervention has potential for improving the sleep and wellbeing of older people at risk or diagnosed with dementia. The mixed-methods approach and use of case studies including both care recipients and care partners was novel, allowing investigation into the context of conducting such a study in the NZ community. The protocol was considered feasible and suggestions for future trials were obtained. The small heterogeneous sample limited the ability to measure the clinical effectiveness of the interventions, or to determine the characteristics of those most likely to benefit. However, some pairs did report benefits to their sleep and waking wellbeing. In other cases, a rapid deterioration of health masked any possible improvements or prevented participants from being able to complete the trial. These mixed findings are concordant with previous research with people living in institutions or the community, using both cross-sectional studies and randomised controlled trials [27]. They are representative of the clinical reality of families affected by dementia.

The interventions offer a low-risk, drug-free option for managing dementia-related sleep disturbances. They were designed to address well-defined physiological and psychosocial processes affecting sleep and waking function (most notably stabilising the function of the circadian master clock) [6, 7]. The protocol in the present study differed from previous community-based trials (e.g. 13, 28) in that bright light was recommended at mid-morning, using a higher intensity and for a shorter duration. The exercise component also had a recommended time frame. Delivering the interventions this way was considered more suitable for the participants, since dementia-related symptoms and behaviours often exacerbate towards evening, which is also a time of high care demands [6]. The timing of the light and exercise exposures was also anticipated to minimise the likelihood of the interventions having an arousing effect on the care recipient before bedtime [11, 12].

Compared to previous community-based studies [13, 28], the present sample of care recipients had more varied types of dementia-related impairment. They also had less problematic sleep at baseline, as indicated by actigraphic and subjective reports as well as higher baseline light exposure (more than 2000 lux). Similarly, the sample of carers had relatively good actigraphic sleep compared to those in previous samples. However, their subjective ratings indicated a high prevalence of sleep problems which was more comparable [8]. Discrepancies between subjective and objective sleep data of carers is not unusual and likely associated with the psychosocial impact of caring on the symptoms of insomnia [8, 29]. Carers in the present sample appeared to have reduced likelihood of anxiety or depression compared to previous samples of NZ carers [25] which may have contributed to different sleep outcomes.

Variable improvement in sleep problems associated with MCIoD may be attributable to a range of factors. These include variability in comorbid diseases and demographic factors, the fluctuating nature of sleep and dementia-related behaviours, as well as response bias, possible placebo effects, and the impact of researcher support. Comparisons between studies are also confounded by differences in sleep measures and intervention characteristics. The course of cognitive deterioration and factors necessary for stable sleep timing and efficiency (including damage to the eye, pineal gland, and circadian system as well as presence of another primary sleep disorder) are also expected to affect intervention effectiveness [27].

Based on this study, it is recommended that future trials are tailored towards care recipients who have limited access to bright light and physical activity, as well those with poor sleep status at baseline. A larger randomised controlled trial would enable greater monitoring of the impact of the intervention while controlling for biases. The Inclusion of qualitative elements in such trials is recommended to strengthen our understanding of the psychosocial elements of sleep health with ageing, MCI, and dementia as well as providing a person-centred perspective on the effectiveness of the interventions in this highly variable population.

Considering the variation between families affected by dementia as well as issues with recruitment, compliance, and attrition in the present study the findings presented here cannot be generalised to all families affected by MCIoD. With appreciation for such diversity and challenges, future trials may benefit from a more personally tailored approach to engagement and delivery. This may also reduce research load on the participants. This could be achieved through using co-designed research methods and customised interventions, automated rather than diary-assisted scoring of actigraphy data (removing the need for keeping dairies) [30], and delivering interventions via primary healthcare or pre-existing home care services with a support person to facilitate uptake. Research of this nature are now underway within a broad research team in NZ (including the first author). Supported by the Ageing Well National Science Challenge, “Ageing Well through Eating, Sleeping, Socialising and Mobility Programme (AWESSoM)” will assess and support key pillar of health non-pharmacologically within at-risk groups of older people living in New Zealand including older Māori, Pacific Islanders, and care home residents.

## Conclusion

With a rapidly ageing population, increases in New Zealanders affected by dementia are projected. Many are supported at home informally via family. This model is becoming increasingly critical for regulating the costs to the healthcare system as well as for facilitating older New Zealanders to live well with advancing age and dementia [5]. This study indicates that a combined non-pharmaceutical intervention for sleep is feasible for people with MCIoD and their family carers at home, with limited research burden. While the effect of the intervention was variable at the group level, some individuals reported improvements to sleep and waking status. We conclude that, given the lack of options for treating dementia, the intervention has potential for improving the sleep and wellbeing of older people at risk or diagnosed with dementia and further trials are warranted.

## Data Availability

The protocol, study documents, and datasets produced and analysed during the current study are available from the corresponding author on reasonable request. In alignment with the Health and Disability Ethics Committee of NZ regulations, data are stored for 10 years in electronic and paper form in secure locations at the Sleep/Wake Research Centre and will be unavailable after July 2022.
